# Experiences of households using integrated malaria prevention in two rural communities in Wakiso district, Uganda: a qualitative study

**DOI:** 10.1186/s12936-016-1369-4

**Published:** 2016-06-07

**Authors:** David Musoke, George Karani, Rawlance Ndejjo, Peter Okui, Miph Boses Musoke

**Affiliations:** Department of Disease Control and Environmental Health, School of Public Health, Makerere University College of Health Sciences, Kampala, Uganda; Cardiff School of Health Sciences, Cardiff Metropolitan University, Cardiff, Wales, UK; National Malaria Control Programme, Ministry of Health, Kampala, Uganda; School of Sciences, Nkumba University, Entebbe, Uganda

**Keywords:** Malaria prevention, Integrated approach, Long-lasting insecticidal nets, Window and ventilator screening, Mosquito breeding sites, Uganda

## Abstract

**Background:**

The integrated approach to malaria prevention which advocates use of several methods in a holistic manner is being explored to complement existing strategies. A pilot project that promoted integrated malaria prevention established 40 demonstration households using the approach. As part of impact evaluation of the project 2 years after implementation, the experiences of these households using integrated malaria prevention were assessed.

**Methods:**

A qualitative cross-sectional survey was carried out in Wakiso district, Uganda which involved 40 in-depth interviews among households implementing integrated malaria prevention. The study assessed practices on malaria prevention, benefits and challenges of using integrated malaria prevention, preference of malaria prevention methods, and impact of the demonstration households on the community. Thematic analysis was employed using Atlas ti software.

**Results:**

The households continued to use many of the malaria prevention methods in the integrated approach including sleeping under long-lasting insecticidal nets, screening in windows and ventilators, removing mosquito breeding sites, and closing of doors early in the evenings. The major benefits reported from using integrated malaria prevention were reduction in mosquito populations in their houses and less occurrence of malaria particularly among children. Although several community members learnt about and admired various malaria prevention methods from the demonstration households especially screening in windows and ventilators, the majority could not afford to implement some of them due to lack of resources. The main challenge established in using integrated malaria prevention was the much time required to implement the several methods some of which had to be done regularly such as early closing of windows. In addition, complacency had led to some households not utilizing a number of methods in the integrated approach because of using others.

**Conclusion:**

Use of the integrated approach to malaria prevention benefited the demonstration households mainly through observed reduction in mosquitoes indoors and malaria occurrence hence could be promoted in other areas. Other studies to quantify the protective effect of integrated malaria prevention particularly regarding malaria prevalence and contribution of each of the methods are required.

## Background

Malaria has for many years remained a leading cause of morbidity and mortality in sub-Saharan Africa including Uganda with children under 5 years of age most affected. Coverage of long-lasting insecticidal nets (LLINs) has significantly increased globally in recent years, and has contributed to the reduction in malaria in endemic countries [1]. However, indoor residual spraying (IRS), another major global malaria prevention strategy, is not as much widespread [1] despite evidence on its efficacy in preventing the disease [2, 3]. In Uganda, use of insecticide-treated nets (ITNs) in the form of LLINs, and IRS are the most advocated malaria prevention methods as is the case in other malaria endemic countries. The Government of Uganda through the Ministry of Health (MOH) has promoted the use of LLINs nationally through mass distribution campaigns, including to children under 5 years of age and pregnant women attending antenatal care in public health facilities [4]. The MOH recently distributed over 21 million LLINs throughout the country with a target of reducing malaria related deaths [5]. However, IRS has been conducted as a national programme in selected districts in the country mainly in the northern region [4]. Whereas households owning at least one ITN nationally were 60 %, only 7 % had undergone IRS in the previous 12 months [6]. Therefore, these two main malaria prevention methods being used in the country are currently underutilized.

The World Health Organization (WHO) recommends use of integrated vector management for malaria control [7], which has shown promise in contributing to reducing the burden of the disease [8]. However, studies have established that other malaria prevention methods beyond ITNs and IRS such as screening in ventilators (openings on houses that allow in fresh air) and draining stagnant pools of water are hardly being used in Uganda and elsewhere [9, 10]. This has necessitated exploring strategies that combine multiple malaria prevention methods in endemic countries. Indeed, the integrated approach to malaria prevention advocates use of several malaria prevention methods to reduce mosquito populations near homes, limit their entry into houses and prevent bites from them which all contribute to reducing malaria transmission. The methods promoted in the integrated approach include: use of LLINs; screening in windows, ventilators and open eaves; removing mosquito breeding sites such as stagnant water; and closing of windows and doors early in the evenings. These practices are known to individually reduce the occurrence of malaria [11–13].

A pilot project promoting the integrated approach to malaria prevention was implemented in two rural communities in Uganda [14]. This project established 40 demonstration households using the various methods in the integrated approach. The purpose of establishing these demonstration households was to ensure that the community used them to learn about the several malaria prevention methods promoted during the project. For this reason, the households were diversely located in the two study villages so as to ensure that most part of the community had access to at least one of them. Community exposure to the demonstration households was expected to influence their knowledge, attitudes and practices regarding malaria prevention. An impact evaluation was carried out 2 years after end of the pilot project to assess the benefits of the project interventions to the community. This evaluation included an exploration of issues concerning use of the integrated approach among the demonstration households, which are reported in this paper.

To explore the potential of use of integrated malaria prevention in rural communities, it is important to understand the benefits, challenges and other pertinent issues pertaining to using the approach. This is necessary before future studies to quantitatively measure the public health impact of the approach are conducted. Qualitative research methods were used in this study to establish various perspectives of users of integrated malaria prevention from the pilot project. Qualitative research facilitates in-depth understanding of issues [15] pertaining to community practices, which is important in early stages of new public health strategies and interventions [16]. Studies that have assessed integrated vector management for malaria prevention have mainly been quantitative with little evidence on community perceptions [17]. This study qualitatively assessed the experiences of households using integrated malaria prevention as a follow-up of the pilot project that promoted the approach in two rural communities in Wakiso district, Uganda.

## Methods

### Study design

The study was a qualitative cross-sectional survey carried out in Wakiso district, Uganda. A total of 40 in-depth interviews were conducted among households implementing integrated malaria prevention. This study was carried out as part of the impact evaluation of the pilot project which was conducted 2 years after implementation. The evaluation was carried out after 2 years so as to assess the long-term impact of the project interventions as well as establish if the malaria prevention methods promoted were still being used.

### Study setting and participants

The pilot project that promoted the integrated approach to malaria prevention was implemented in Mayanzi zone, Entebbe Municipality and Lukose zone, Ssisa sub-county both in Wakiso district, Uganda. These two rural communities, which predominantly participate in agriculture, small-scale trade, brick making and fishing, are located in the central region of the country. Most of the houses in the area are permanent and made of bricks. The houses have windows and ventilators hence the feasibility to screen them against mosquito entry. The project established 40 demonstration households that started using integrated malaria prevention. These households, 20 in each study area, were selected by the respective village leaders. During selection, it was ensured that the households had at least one child under 5 years of age or a pregnant woman as the groups most affected by malaria. In addition, the location of households was considered to ensure that they were well distributed hence the entire community had access to them. The demonstration household members were trained on the use of several malaria prevention methods in an integrated manner to prevent malaria. These households were then provided with LLINs, and had their windows and ventilators screened to prevent mosquito entry. The few houses among the selected households that had open eaves were also screened. The number of LLINs provided, which ranged from two to six, depended on household size and available nets in use. The other malaria prevention methods in the integrated approach such as removing mosquito breeding sites, and early closure of doors and windows were implemented by respective households. More details on the pilot project can be found in an earlier publication [13]. Participants in the study were heads of the 40 demonstration households. Where household heads were not found during data collection, other responsible adults were used.

### Data collection

The 40 participants from the demonstration households underwent in-depth interviews to establish their experiences of using the integrated approach to prevent malaria. These interviews were unscheduled to prevent households carrying out any activities specifically in anticipation of data collection. The in-depth interview guide used had questions related to practices on malaria prevention, benefits and challenges of using integrated malaria prevention, preference of malaria prevention methods, and the impact of the demonstration households to the community. The guide developed in English was translated to *Luganda*, the local language mostly used in the study area. Data was collected in *Luganda* by the principal investigator with support of a research assistant who recorded all proceedings of the interviews. The interviews were conducted in an ideal location suggested by the participants with no other household members allowed near the data collection activity. The average duration of the in-depth interviews was 45 min.

### Data analysis

All in-depth interviews were tape recorded and later transcribed verbatim in *Luganda* by the research assistant. The principal investigator then read the transcriptions to ensure they were a true representation of the data collected. Minor editing to the transcripts was done at this stage. Once the transcripts had been validated, they were translated to English by the research assistant and verified by the principal investigator. Codes were developed from the study objectives and transcribed data for use in analysis. Using Atlas ti version 6.0.15 qualitative data analysis software, the transcripts were coded. The coded transcripts were reviewed by two researchers who then used them to adequately categorize the data. Using thematic analysis, the categorized data was used to generate themes which are employed to present the major findings from the study.

### Ethical considerations

The study received ethical approval from Makerere University School of Public Health Higher Degrees, Research and Ethics Committee. The study was also approved and registered by the Uganda National Council for Science and Technology which is a legal requirement in Uganda. The local leaders in the two villages were informed about the study and provided permission to collect data in the area. All participants provided written informed consent before their involvement in the study.

## Results

Of the 40 participants, 28 were female of whom the majority were spouses to household heads. Most of the participants were staying with their spouses (married or cohabiting) and involved in agriculture while others operated small business at or near their homes. Although all participants had attended school, they had either stopped in primary or secondary levels with none having reached tertiary institutions or university. The results of this study are presented under the five main themes generated: malaria prevention practices of demonstration households; benefits of using integrated malaria prevention; use of the households for community demonstration; challenges of using integrated malaria prevention; and, opinion regarding scaling up integrated malaria prevention.

### Malaria prevention practices of demonstration households

**Fig. 1:**
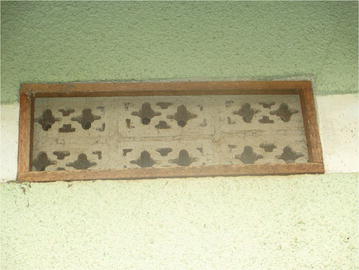
A ventilator with screening on one of the demonstration houses

**Fig. 2:**
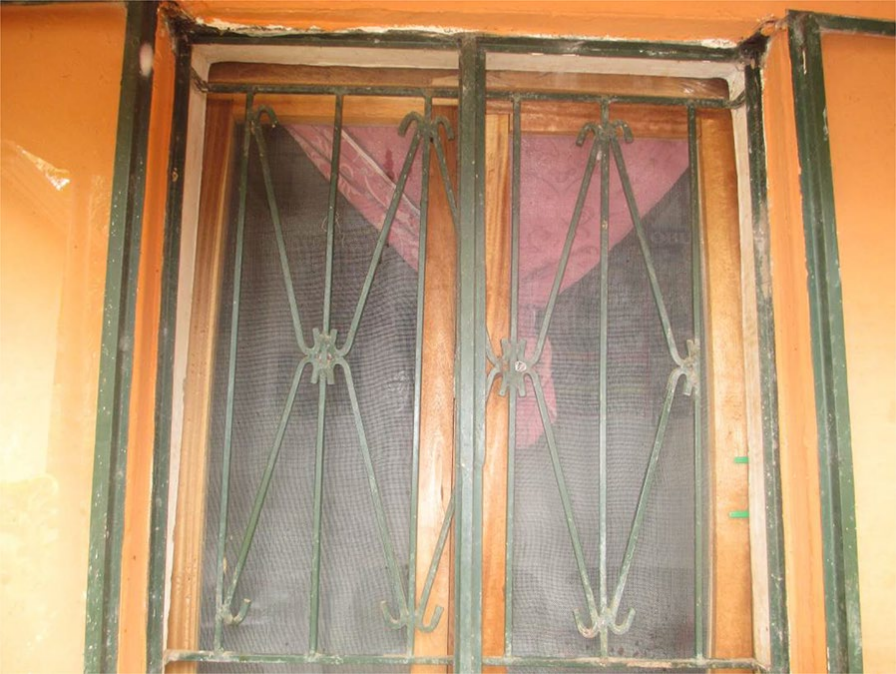
A window with screening on one of the demonstration houses

**Fig. 3:**
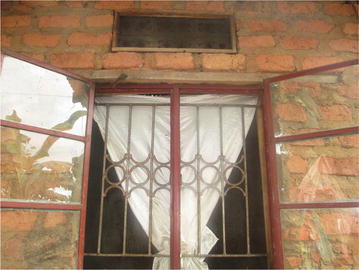
A window and ventilator with screening on one of the demonstration houses

The demonstration households continued to use many of the malaria prevention methods in the integrated approach that they were trained during the pilot project. These included sleeping under LLINs, screening in windows and ventilators (Figs. 1, 2, 3), removing mosquito breeding sites, and closing of doors early in the evenings.

“We utilize those methods that we were taught during the training on malaria prevention for example closing doors and windows at 5.00 pm, and draining of stagnant pools of water. We also use the mosquito nets that we were provided with so as to prevent mosquito bites while sleeping.” Demonstration household 6, Mayanzi village

“Although we have mosquito nets, the practice of closing doors and windows early in the evenings is something we do every day. This practice is easy for us given that even when an adult is not at home, the children are knowledgeable about the recommended time for closing the doors and windows on the house which they do promptly.” Demonstration household 16, Lukose village

Regarding preference of the two interventions that the project provided to the households (LLINs and screening in windows and ventilators), mixed responses were observed. Some of the participants were in favour of LLINs because of their known efficacy in malaria prevention. However, it was noted that there were situations where LLINs could not be used such as when they are insufficient for entire households or disliked for various reasons such as discomfort due to increased heat. In such scenarios, screening in windows and ventilators was seen to be more beneficial especially to all household members if external doors on houses were closed at an appropriate time.

“I think sleeping under a mosquito net works better because there are some days when I may delay to close the doors and mosquitoes enter the house. However, if I sleep under my net on such a day, there is no risk of mosquitoes biting me while asleep.” Demonstration household 19, Lukose village

“In my view, proofing in windows and ventilators would be better than mosquito nets. You may have enough mosquito nets for your household members but not for your visitors and their children. In such cases, the screens in windows and ventilators would still keep the mosquitoes away from entering the house and protect all the people including those without mosquito nets.” Demonstration household 2, Mayanzi village

### Benefits of using integrated malaria prevention

The study participants reported several benefits for their households ever since they started using integrated malaria prevention. One of the major benefits observed was the reduction in mosquito populations in their houses ever since the various interventions notably screening in windows and ventilators was implemented.

“We have benefited a lot from the interventions the project implemented for us. The various barriers we use in the home have been effective in preventing us from encountering mosquitoes. The screens in the windows and ventilators ensure mosquitoes are kept away and if by any chance they enter the house, they cannot pass through the nets we use at night hence we do not get bitten by mosquitoes.” Demonstration household 8, Mayanzi village

“We have really experienced changes in the mosquito population in our home. Previously, whenever I entered the house, mosquitoes could be hovering around the children but nowadays, I no longer see any. I can only attribute this change to the nets that were installed in the windows and ventilators. We also endeavour to close the doors and windows early in the evening.” Demonstration household 20, Mayanzi village

The participants reported (qualitatively) that occupants of their households particularly children suffered less from malaria ever since they started using the integrated approach. This led to less expenditure on malaria treatment, reduced visits to health facilities, and fewer days of children missing school. These outcomes were attributed to the interventions they were using which were seen to have generally led to improved family wellbeing.

“There is a significant difference in the occurrence of malaria in our household. Before I started using those methods, I could have a child suffering from malaria almost every month which made me spend a lot of money buying drugs and taking these children to health centres. However, when I started using mosquito nets and proofing in windows among other practices, malaria is now very rare in my household. In fact, only one child has suffered from malaria in the last 5 months.” Demonstration household 1, Lukose village

“There is certainly a difference now. Previously, my children would get recurrent episodes of malaria every month but nowadays, they take a very long time without falling sick. They can go to school consistently for a whole term and no longer have to miss classes due to malaria which is different from the situation before the project. Actually, ever since we were given nets and we implemented the other interventions, I have personally spent over a year without going to the health facility.” Demonstration household 10, Mayanzi village

### Use of households for community demonstration

The study established that several community members learnt about various malaria prevention methods from the demonstration households especially screening in windows and ventilators. Some individuals showed interest in having their households also benefit from interventions implemented in demonstration households. However, it was established that although many households had appreciated the various interventions, the majority could not afford to implement some of the methods such as screening in ventilators as they lacked the required resources mainly financial.

“Many community members who saw how we had benefitted from the project kept asking how we got the proofing in the windows and whether they could also get a similar opportunity. We told them that we were selected by the local leaders to be part of a project and advised them to buy the screens and have them installed on their houses. However, many of them often told us that they did not have the money to do so.” Demonstration household 2, Lukose village

“As my house is near the main road, many people usually ask where I got the knowledge of putting proofing in windows and ventilators from. I then tell them about the project and how such methods can be used to control mosquitoes in their households as well. I normally advise them to also install the nets if they can but very few people in this village have the means. When one of my sisters came to visit me, she was so impressed with how the screens had been fitted. She told me that she would do the same when she goes back to her home.” Demonstration household 11, Mayanzi village

In addition to the screening in windows and ventilators preventing mosquitoes from entering houses among demonstration households, it was established that the houses looked better than they did before. This was noted by both members of the demonstrations households and the general community.

“Very many people have admired our house and they usually ask how we got screening in our windows and ventilators. I usually tell them that some people promoting malaria prevention brought us trainings where we learnt about several methods and then 20 households were selected in which they installed them so that other community members could learn and replicate them in their households. Recently when we had a party here, many community members said our house now looked better and they really appreciated it.” Demonstration household 19, Mayanzi village

### Challenges of using integrated malaria prevention

From experience of the households, it was evident that many of the methods required to be implemented continuously which was time consuming. This included early closing of doors and windows, and removing potential mosquito breeding places. In addition, some of the interventions were only effective if households observed certain practices. As an example, screening in windows and ventilators would only significantly limit mosquito entry if doors were kept closed in the evenings and throughout the night.

“If one takes it for granted that they have proofing in windows and ventilators and do not bother to close the doors before evening, mosquitoes will enter the house and may bite you even before going to bed where you could have a mosquito net. This practice of closing doors early has to be done every day irrespective of who is at home.” Demonstration household 9, Lukose village

It was established that since the integrated approach involved using several malaria prevention methods, complacency had led to households not utilizing some of the methods because of using others. For example, a few households had stopped sleeping under LLINs because of having screening in windows and ventilators on their houses.

“Ever since my windows and ventilators were screened, mosquitoes no longer enter my house. For that reason, I see no need to sleep under a mosquito net anymore. In fact, I have now used the net that was given to me by the project to act as a pillow.” Demonstration household 13, Mayanzi village

“We don’t use mosquito nets since they bring discomfort in form of heat in the night. Without a net, I sleep comfortably with my grandchildren. We only ensure that we close the doors early as we were advised. I think the screens installed in the ventilators and windows have been the most beneficial intervention since even my older children who do not sleep under mosquito nets for the same reasons as me don’t get malaria.” Demonstration household 4, Lukose village

In addition, it was found out that some of the methods in the integrated approach were also the responsibility of other households as well as the community such as removing mosquito breeding sites. It therefore required that other households were aware of and implementing the various methods so as to have a greater impact in the community.

“In this village, we have several large ponds of stagnating water arising from sand mining in addition to small pockets of water that collects at homes when it rains. Therefore, even when I drain stagnant water from my compound and those ponds are not addressed, mosquitoes will still breed in the community. In addition, other households also need to prevent water stagnating in their compounds for example by filling any depressions in the ground.” Demonstration household 6, Lukose village

### Opinions regarding scaling up integrated malaria prevention

The demonstration households generally supported use of the integrated approach in other homes in future due to the benefits they experienced for over 2 years. It was stressed that more sensitization was needed to make the community more aware of the various available malaria prevention methods so as to positively influence their practices.

“I fully support the integrated approach because these methods are very effective in keeping mosquitoes away and preventing them from entering the house. Even when you open the door and a few of them sneak in, they will not be able to bite you since you will sleep under your mosquito net further showing the importance of many barriers. I would support other households to also receive and use the interventions as my family has benefitted a lot from them.” Demonstration household 8, Lukose village

“It is generally easy to implement the several malaria prevention methods. It doesn’t help having a bushy compound where mosquitoes will hide and continue entering your house. I also don’t see anything hard in slashing overgrown grass or removing bottles from the compound to prevent mosquito breeding. More households should be taught and supported to use these methods to prevent suffering from malaria.” Demonstration household 11, Lukose village

## Discussion

Preventing the occurrence of malaria in endemic communities is crucial to reduce the burden of the disease among affected countries particularly in sub-Saharan Africa. This study established that malaria prevention practices in the integrated approach that the demonstration households had been trained during the pilot project were largely still being used. This is a promising finding which provides optimism that continued campaigns aimed at increasing awareness on malaria prevention methods are likely to result in improved preventive behaviour. Indeed, it has been shown that empowering communities with knowledge on malaria prevention through health education can lead to improved practices [18–20]. However, as the current global and national malaria prevention efforts have focused on ITNs and IRS, little attention has been given to promoting other malaria prevention practices despite evidence of these methods individually contributing to reducing the burden of malaria [11–13]. The experiences of this pilot project demonstrate that use of several malaria prevention methods by households could be increased if multiple methods (beyond ITNs and IRS) are promoted by various stakeholders including ministries responsible for health.

The main benefits demonstration households realized from using the integrated approach were reported reduction in mosquitoes in their houses and malaria occurrence. This finding shows that use of several malaria prevention methods is likely to have more impact than single methods as has been demonstrated in other studies [21-23]. However, these studies that have measured the actual reduction in the prevalence of malaria due to use of multiple methods have mainly focused on use ITNs and IRS. Given the promise shown in this pilot project, there is need to carry out more rigorous investigations such as randomized community trials to quantify the actual benefits of the integrated approach among households particularly in terms of malaria prevalence.

The community was able to learn about the methods in the integrated approach being used by the demonstrations households particularly screening in windows and ventilators to prevent mosquito entry. However, the study established that majority of the population could not afford to have such screening installed on their houses. It is well known that poverty in developing countries such as Uganda affects malaria prevention practices including use of ITNs [24]. Indeed, the majority of ITNs owned nationwide have been provided for free by the Government through the Ministry of Health [4]. This lack of resources is, therefore, expected to negatively affect the use of methods in the integrated approach that require funds such as screening in windows and ventilators. However, some of the practices such as early closing of doors and windows which do not require financial resources should be implemented by households irrespective of other malaria prevention methods.

The demonstration households reported that implementing the several practices in the integrated approach was time consuming hence could limit use of all the methods. Indeed, use of multiple methods particularly environmental management can be work-intensive [25]. This concern is important to consider while promoting the integrated approach on a large scale. Although it may not be realistic for all households to implement all the methods in the integrated approach, use of as many methods as may be possible is likely to be more beneficial than using a single method. Therefore, whereas promoting the integrated approach in communities may not necessarily lead to use of all the methods, it would encourage utilization of as many practices that each household can manage. This includes simple measure such as removal of mosquito breeding sites, as well as use of ITNs and screening in windows and ventilators. Early closing of doors and windows in evenings is also practical. These practices are likely to contribute to reduction in the burden of malaria as observed in this study.

A major concern from the study on the experiences of using the integrated approach was complacency resulting in non-use of certain methods in favour of others. This was mainly seen for discarding ITNs due to having screening in windows and ventilators. During the training conducted among the demonstration households on the integrated approach in the pilot project, it was stressed that the various practices were complementing each other and not replacing any method. It is also important to note that different malaria prevention methods have varying levels of efficacy regarding malaria prevention. This requires that studies to measure the efficacy of other methods for malaria prevention are conducted. This is important as use of ITNs and IRS is sometimes compromised due to financial, social and cultural factors [26, 27]. Such research would enable the ranking of the most and least effective methods in malaria prevention among those in the integrated approach. In addition, the finding on complacency needs more community sensitization in future work to promote the integrated approach to malaria prevention, which even in its infancy is generating considerable interest in communities [14].

It was evident from this study that some interventions in the integrated approach such as removal of mosquito breeding sites are not only household but community responsibility. Indeed, demonstration households whose neighbours did not implement these measures felt that it negatively affected their malaria prevention. Anopheles mosquitoes that transmit malaria are known to travel long distances, up to two kilometres from breeding sites [28]. It is, therefore, necessary to reduce such breeding places in entire villages and not merely households. Future promotion of the integrated approach should ensure that interventions targeting mosquito breeding places are not only implemented at household level but also within entire communities so as to have better impact in the areas. Larviciding, which has been recommended for malaria prevention by the WHO in communities where breeding sites are few, fixed and findable [29], could be used for this purpose.

From the experiences of demonstration households in using the integrated approach, they generally supported scaling up of the strategy. This was due to the benefits that they observed while implementing the approach. Such pilot projects are important in generating evidence while exploring new public health interventions [30–32]. Due to the known benefits of the various methods in preventing malaria as well as the advantages of integrated malaria prevention approaches [33], promoting use of multiple practices at households and in communities is necessary.

A limitation of this study is that since the demonstration households had received certain interventions during the project, this could have influenced the responses provided in this study. However, given that the use of multiple interventions to prevent malaria in endemic communities especially in Uganda is low, the design of this study was justified for the pilot project. Future studies among households using integrated malaria prevention without having received external support are required. However, such studies may only be feasible once there is increased use of integrated malaria prevention in communities.

## Conclusion

The use of the integrated approach to malaria prevention benefitted the demonstration households mainly through observed reduction in mosquitoes indoors and malaria occurrence hence could be promoted in other areas. Other studies to quantify the protective effect of the integrated approach particularly regarding malaria prevalence and contribution of each of the methods are required.

## Abbreviations

ITN: insecticide-treated mosquito nets
LLIN: long-lasting insecticide net
MOH: Ministry of Health, Uganda
IRS: indoor residual spraying
